# Associations Between the Kynurenine Pathway, Proinflammatory Cytokines, and Brain-Derived Neurotrophic Factor in Hospitalized Patients With Chronic Schizophrenia: A Preliminary Study

**DOI:** 10.3389/fpsyt.2021.696059

**Published:** 2021-07-28

**Authors:** Naomichi Okamoto, Tomoya Natsuyama, Ryohei Igata, Yuki Konishi, Hirofumi Tesen, Atsuko Ikenouchi, Reiji Yoshimura

**Affiliations:** Department of Psychiatry, University of Occupational and Environmental Health Japan, Kitakyushu, Japan

**Keywords:** schizophrenia, kynurenine pathway, kynurenine, quinolinic acid, inflammatory cytokines, BDNF

## Abstract

**Purpose:** The kynurenine (Kyn) pathway may play a role in the pathophysiology of schizophrenia. This pathway shows crosstalk with proinflammatory cytokines, including interleukin-1β (IL-1β), IL-6, and tumor necrosis factor-α (TNF-α), and/or brain-derived neurotrophic factor (BDNF). Moreover, Kyn metabolites affect neurotransmission and cause neurotoxicity. To date, the influence of the Kyn pathway on proinflammatory cytokines and BDNF remains to be fully elucidated. The aim of this study was to investigate the relationships of the Kyn pathway with proinflammatory cytokines, BDNF, and psychiatric symptoms in patients with schizophrenia.

**Methods:** Thirty patients with schizophrenia and ten healthy control participants were recruited for this study. All patients were diagnosed with schizophrenia using the Diagnostic and Statistical Manual for Mental Disorders, Fifth Edition (DSM-5). The healthy controls were those who did not fulfill any of the diagnostic criteria in the DSM-5. The serum levels of Kyn and its metabolites, proinflammatory cytokines, and BDNF were measured in patients with schizophrenia and healthy controls. Patients with schizophrenia were also assessed for psychiatric symptoms using the Positive and Negative Syndrome Scale (PANSS).

**Results:** Patients with schizophrenia and healthy controls showed no significant differences in the levels of Kyn and its metabolites, proinflammatory cytokines, and BDNF. A significant positive correlation was found between the serum levels of TNF-α and Kyn (*r* = 0.53, *p* = 0.0026) and the Kyn/tryptophan (Trp) value (*r* = 0.67, *p* = 0.000046) in the schizophrenia group, but not in the healthy control group.

**Conclusion:** TNF-α affects the Kyn pathway in patients with chronic schizophrenia, but not in the healthy individuals, although serum TNF-α levels showed no difference between the two groups. Associations between the Kyn pathway and the levels of proinflammatory cytokines and BDNF or psychotic symptoms might be complicated in hospitalized patients with chronic schizophrenia.

## Introduction

Although the pathophysiology of schizophrenia is currently unclear, inflammation has been suggested to play an important role in the pathophysiology (1, 2). In addition, there is growing evidence of an interaction between inflammation and the kynurenine (Kyn) pathway in schizophrenia (3). The Kyn pathway is regulated by the immune system, and the decomposition of tryptophan *via* the Kyn pathway is activated by proinflammatory cytokines. The Kyn pathway is also considered to crosstalk with the immune system, proinflammatory cytokines, and neurotrophic factors (4, 5). Tryptophan (Trp) is degraded to Kyn, which is catabolized to either kynurenic acid (KynA) *via* kynurenine aminotransferases or to 3-hydroxykynurenine (3-HK) *via* kynurenine 3-monooxygenase and finally to quinolinic acid (QA). Proinflammatory cytokines, including interleukin-1β (IL-1β), IL-6, and tumor necrosis factor-α (TNF-α), are thought to contribute to the pathogenesis of psychiatric symptoms in schizophrenia by Kyn pathway activation.

Kyn metabolites affect neurotransmission and cause neurotoxicity. Several studies have indicated that proinflammatory cytokines and the Kyn pathway in the blood are dysregulated in patients with schizophrenia. Increase in the plasma levels of proinflammatory cytokines, such as IL-1β, IL-6, and TNF-α, have been consistently reported in patients with schizophrenia (6). C-reactive protein (CRP) levels have also been reported to be elevated in patients with schizophrenia (7). Thus, these proinflammatory cytokines may accelerate the Kyn pathway (8), which may be related to changes in neurotrophic factors, which are associated with the symptoms of schizophrenia. According to the review (9), the Kyn pathway of Trp degradation generates several neuroactive compounds. KynA is an N-methyl-d-aspartate (NMDA) and alpha 7 nicotinic receptor antagonist. The KynA hypothesis of schizophrenia is based on the fact that KynA blocks glutamate receptors and is elevated in schizophrenia. KynA regulates glutamatergic and dopaminergic neurotransmission and elevated brain levels appear associated with psychotic symptoms and cognitive impairments in schizophrenia. Contributing to enhanced production of KynA, the expression and enzyme activity of kynurenine 3-monooxygenase (KMO) are reduced in schizophrenia. There were several works by Engberg and Erhardt. The authors reported cerebrospinal fluid (CSF) levels of Kyn, and KynA in schizophrenia patients (10, 11). They also reported CSF levels of Kyn and KynA were elevated in patients with chronic schizophrenia, indicating the idea of brain immune activation in patients with schizophrenia, which suggested that IL-6 induces the Kyn pathway, leading to increased production of the NMDA receptor antagonist KynA in patients with schizophrenia (12). A recent report demonstrated that proinflammatory cytokines are involved in dorsolateral prefrontal cortex volume loss and attention impairment *via* the Kyn pathway (13). However, the associations among these components are complicated and controversial. Recent review reported the following findings, (1) schizophrenia patients with prescribed antipsychotic drugs had significantly higher Kyn levels compared with controls; (2) higher Kyn levels in CSF, lower plasma Kyn levels compared with controls; (3) the Kyn levels were higher in schizophrenia patients after treatment with antipsychotic drugs compared with baseline (14). Therefore, the correlations among proinflammatory cytokines, neurotrophic factors, and the Kyn pathway and their possible relevance to schizophrenic pathophysiology, must be further elucidated.

Thus, the aim of the present study was to investigate the associations of proinflammatory cytokines, including IL-1β, IL-6, TNF-α, and IL-10, a suppressive cytokine, and brain-derived neurotrophic factor (BDNF) with the Kyn pathway, which might be related to the symptomatology of schizophrenia patients. The present study aimed to shed light on how coordination among the Kyn pathway, proinflammatory cytokines, and BDNF may influence the symptomatology in chronic hospitalized schizophrenia patients.

## Methods

### Participants and Ethics Statement

Thirty patients with schizophrenia participated in this study. The patients were recruited from the Komine-Eto Hospital and Shin-moji Hospital. All patients were diagnosed with schizophrenia using the Diagnostic and Statistical Manual for Mental Disorders, Fifth Edition (DSM-5) (15). Exclusion criteria included a history of major neurological disease, uncontrolled major medical illness, epilepsy, cerebrovascular accident, head trauma with cognitive sequelae, and intellectual disability. All patients with schizophrenia were taking antipsychotic medications. The control group of healthy controls did not currently have a DSM-5 applicable psychiatric diagnosis, nor did they have a family history of psychosis. All participants provided verbal and written informed consent. The research protocol was approved by the Ethics Committee of the University of Occupational and Environmental Health (Approval Number: UOEHCRB19-024).

### Clinical Assessment and Blood Sampling

Patients with schizophrenia were assessed for clinical and neuropsychiatric symptoms using the Positive and Negative Syndrome Scale (PANSS) (16) and the Drug Induced Extra-Pyramidal Symptoms Scale (DIEPSS) (17). Blood samples were collected between 7 and 9 a.m. before breakfast. Participants fasted and rested for at least 30 min prior to blood collection.

### Measurement of Metabolites of the Kyn Pathway

The samples obtained at the University of Occupational and Environmental Health were transferred to Human Metabolome Technologies Inc. (HMT; Tsuruoka, Japan), where each 50-μL sample was mixed with 450 μL of methanol containing internal standards (10 μM) and vortexed. Chloroform (500 μL) and Milli-Q water (200 μL) were added, mixed thoroughly, and centrifuged (2,300 × g, 4°C, 5 min). The water layer (400 μL) was filtered through a 5-kDa-cutoff filter (ULTRAFREE-MC-PLHCC; HMT, Yamagata, Japan) to remove macromolecules. The filtrate was centrifugally concentrated and resuspended in 50 μL of ultrapure water immediately before measurement. The compounds were measured in the cation and anion modes of a capillary electrophoresis-Fourier transform mass spectrometry (CE-FTMS)-based metabolome analysis system under conditions 1–3. The samples were diluted for the measurements to improve the quality of the CE-FTMS analysis. Metabolome measurements were conducted at HMT by using CE-time-of-flight mass spectrometry (TOFMS) in an Agilent CE System equipped with the Agilent 6210 time-of-flight mass spectrometer, the Agilent 1100 isocratic HPLC pump, the Agilent G1603A CE-MS adapter kit, and the Agilent G1607A CE-ESI-MS sprayer kit (Agilent Technologies, Waldbronn, Germany). The system was controlled by Agilent G2201AA ChemStation software version B.03.01 for CE (Agilent Technologies, Waldbronn, Germany). The metabolites were analyzed using a fused silica capillary (50 μm internal diameter ×80 cm total length), with commercial electrophoresis buffers (Solution ID: H3301-1001 for cation analysis, H3302-1021 for anion analysis; HMT) as the electrolyte. The sample was injected at a pressure of 50 mbar for 10 s (~10 nL) for cation analysis and 25 s (~25 nL) for anion analysis. The spectrometer was scanned from 50 to 1,000 m/z. Other conditions were as described previously (18–20). Peaks were extracted using the automatic integration software MasterHands (Keio University, Tsuruoka, Japan) to obtain peak information, including m/z, migration time for CE-TOFMS measurement (MT), and peak area values (21). Signal peaks corresponding to isotopomers, adduct ions, and other product ions of known metabolites were excluded, and the remaining peaks were annotated with putative metabolites from the HMT metabolite database based on their MTs and m/z values, as determined from TOFMS. The tolerance range for the peak annotation was configured at ±0.5 min for MT and ±10 ppm for m/z. In addition, peak areas were normalized against those of the internal standards, and the resultant relative areas were further normalized by the sample amount. Serum KynA levels were under limit of detection.

### Measurement of Serum Inflammatory Cytokine and BDNF Levels

Samples were collected at the University of Occupational and Environmental Health and transferred to SRL Inc. (SRL: Kitakyushu, Japan). Serum cytokine (IL-1β, IL-6, IL-10, TNF-α) and BDNF levels were measured using the sandwich enzyme immunoassay technique. A monoclonal antibody specific for humans was pre-coated onto a microplate. Standards and samples were pipetted into the wells and any IL-1β, IL-6, IL-10, TNF-α, and BDNF present were bound by the immobilized antibody. After washing away any unbound substances, a biotinylated polyclonal antibody specific for humans was added to the wells. After a wash to remove any unbound antibody-biotin reagent, enzyme-linked streptavidin was added to the wells. After washing away any unbound streptavidin-enzyme reagent, a substrate solution was added to the wells and color developed in proportion to the amount of IL-1β, IL-6, IL-10, TNF-α, and BDNF bound in the initial step. Color development was stopped, and the intensity of the color was measured. Serum levels of IL-1β, and IL-10 were under limit of detection.

### Measurement of Serum hsCRP Levels

The high-sensitivity CRP (hsCRP) levels were measured using a commercially available enzyme-linked immunosorbent assay (MSD, Rockville, MD, USA).

### Statistical Analyses

All statistical analyses were performed using the EZR software version 1.50, a modified version of R Commander designed to perform statistical functions frequently used in biostatistics (22). After confirming the normal distribution and equality of variance, continuous variables were analyzed using Student's *t*-test, Welch's test, and Mann-Whitney U test. Fisher's -test was used to analyze nominal variables. Spearman's rank correlation coefficient was used to examine the relationship among the levels of metabolites in the Kyn pathway, including their ratios (Trp, Kyn, 3-HK, QA, 5-hydroxytryptamine [5-HT], Kyn/Trp, 5-HT/Kyn, 3-HK/Kyn, QA/Kyn), and the levels of proinflammatory cytokines (TNF-α, IL-6), hsCRP, BDNF and PANSS. The correlations between the data were expressed by correlation table. Positive correlations are shown in red, negative correlations are shown in blue, and the strength of the correlation is expressed in terms of concentration. A two-tailed test was used, and a *p*-value <0.05 was defined as a statistically significant difference. Dates were expressed mean(standard deviation) or median [interquartile range]. Figures that showed statistically significant differences in the correlation table were drawn. The regression line was drawn based on the least squares method. One schizophrenia patient had outlier data for CRP (7.2 mg/dL) and IL-6 (24 pg/mL) levels; therefore, we excluded these data.

## Results

### Background Characteristics

Thirty patients with schizophrenia and ten healthy participants were included in this study. The background and clinical characteristics of the patients with schizophrenia and healthy controls are shown (Table 1). No significant difference was observed in the background characteristics between patients with schizophrenia and healthy controls.

**Table 1:** Clinical and demographic characteristics.

	**Schizophrenia**	**Healthy controls**	***p-*value**
	**(*n* = 30)**	**(*n* = 10)**	
Age (years)	48 (9.0)	48 (8.7)	0.99
Sex (males, %)	17 (57%)	5 (50%)	0.73
BMI (kg/m^2^)	23 (3.8)	23 (2.8)	0.89
**PANSS**
PANSS total	95 (13)	–	–
PANSS positive	21 (4.6)	–	–
PANSS negative	26 (4.9)	–	–
PANSS general	48 (8.4)	–	–
DIEPSS	6.3 (3.8)	–	–
CP total	745 (460)	–	–
Disease period	26 (10)	–	–
Antipsychotic drugs	Olanzapine (6 cases)		
	Risperidone (6 cases)		
	Levomepromazine (5 cases)		
	Aripiprazole (5 cases)		
	Clozapine (5 cases)		
	Haloperidol (4 cases)		
	Brexpiprazole (3 cases)		
	Quetiapine (3 cases)		
	Zotepine (3 cases)		
	Asenapine (2 cases)		
	Blonanserin (2 cases)		
	Fluphenazine (1 cases)		

*BMI, body mass index; PANSS, Positive and Negative Syndrome Scale; DIEPSS, Drug Induced Extra-Pyramidal Symptoms Scale; CP total, total dose of chlorpromazine equivalence. Dates were expressed mean(standard deviation)*.

### Serum Levels of the Metabolites of the Kyn Pathway in the Schizophrenia and Healthy Control Groups

We evaluated the serum levels of metabolites of the Kyn pathway (Trp, Kyn, 3-HK, QA, 5-HT, Kyn/Trp, 5-HT/Kyn, 3-HK/Kyn, QA/Kyn) in patients with schizophrenia and healthy controls. No significant differences were observed in the levels of the metabolites of the Kyn pathway between patients with schizophrenia and healthy controls (Table 2).

**Table 2:** Serum levels of the metabolites of the Kyn pathway in the schizophrenia and healthy control groups.

	**Schizophrenia (*n* = 30)**	**Healthy controls (*n* = 10)**	***p-*value**
Trp	1.9 × 10^−1^ (4.5 × 10^−2^)	2.1 × 10^−1^ (3.1 × 10^−2^)	0.11
Kyn	6.0 × 10^−3^ (2.0 × 10^−3^)	6.9 × 10^−3^ (1.3 × 10^−3^)	0.26
3-HK	5.6 × 10^−5^ [4.3 × 10^−5^~8.0 × 10^−5^]	7.1 × 10^−5^ [5.8 × 10^−5^~1.0 × 10^−4^]	0.052
QA	8.6 × 10^−5^ [6.6 × 10^−5^~1.4 × 10^−4^]	1.1 × 10^−4^ [4.6 × 10^−5^~1.4 × 10^−4^]	0.99
5-HT	1.1 × 10^−3^ (5.3 × 10^−4^)	1.0 × 10^−3^ (6.8 × 10^−4^)	0.80
Kyn/Try	0.03 [0.02~0.06]	0.03 [0.02~0.05]	0.93
5HT/Kyn	0.17 [0.094~0.25]	0.12 [0.11~0.20]	0.57
3-HK/Kyn	0.01 [0.01~0.10]	0.01 [0.01~0.02]	0.97
QA/Kyn	0.01 [0.01~0.02]	0.01 [0.008~0.02]	0.98

*Trp, tryptophan; Kyn, kynurenine; 3-HK, 3-hydroxykynurenine; QA, quinolinic acid; 5-HT, 5-hydroxytryptamine; KynA, kynurenic acid. Dates were expressed mean(standard deviation) or median [interquartile range]. The peak areas of the metabolites were normalized against those of the internal standards, and the resultant relative areas were further normalized by the sample amount explained in the Measurement of metabolites of the Kyn pathway. Thus, each figure of Trp, Kyn, 3-HK, QA, 5-HT demonstrated as the ratio comparing with internal standard*.

### Serum Levels of Cytokines, hsCRP, and BDNF in the Schizophrenia and Healthy Control Groups

We also evaluated the serum levels of cytokines (TNF-α, IL-6), hsCRP, and BDNF in patients with schizophrenia and healthy controls (Table 3). No significant difference was observed in serum levels of the cytokines (TNF-α, IL-6), hsCRP, and BDNF between the schizophrenia and healthy control groups. IL-1β and IL-10 were not detected in the samples.

**Table 3:** Serum levels of cytokines, hsCRP, and BDNF in the schizophrenia group and the healthy control.

	**Schizophrenia (*n* = 30)**	**Healthy controls (*n* = 10)**	***p-*value**
IL-6 (pg/mL)	1.5 [0.9~2.1]	1.1 [0.6~2.0]	0.23
TNF-α (pg/mL)	0.9 [0.7~1.0]	0.9 [0.7~0.9]	0.52
hsCRP (mg/dL)	0.029 [0.0070~0.065]	0.030 [0.017~0.065]	0.80
BDNF (pg/mL)	22,000 (7,800)	22,000 (5,000)	0.99

*hsCRP, high-sensitivity C-reactive protein; BDNF, brain-derived neurotrophic factor; IL, interleukin; TNF-α, tumor necrosis factor alpha. Dates were expressed mean(standard deviation) or median [interquartile range]*.

### Relationships Among Metabolites of the Kyn Pathway and Cytokines, hsCRP, and BDNF in the Schizophrenia and Healthy Control Groups

**Figure 1:**
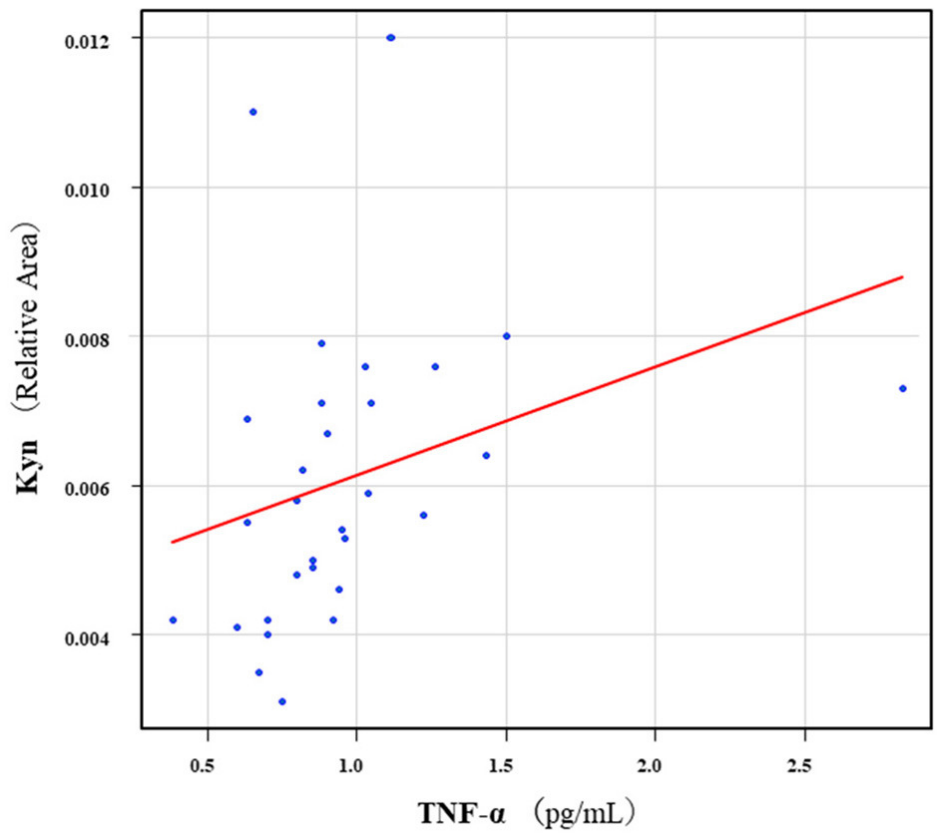
Association between serum TNF-α levels and serum Kyn levels in the schizophrenia group. A significant positive correlation was found between the serum TNF-α levels and the serum Kyn levels (*r* = 0.53, *p* = 0.0026). Relative Area: The peak areas of the metabolites were normalized against those of the internal standards, and the resultant relative areas were further normalized by the sample amount explained in the Measurement of metabolites of the Kyn pathway. Thus, serum Kyn levels demonstrated as the ratio comparing with internal standard.

**Figure 2:**
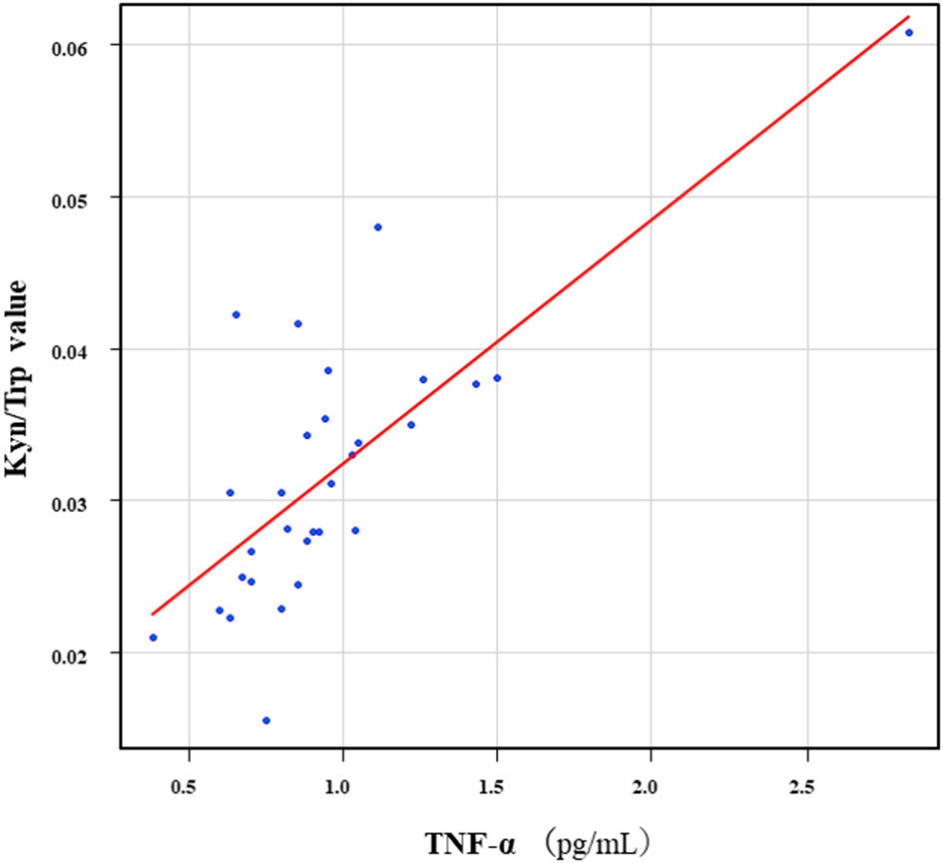
Association between serum TNF-α levels and the Kyn/Trp value in the schizophrenia group. A significant positive correlation was found between the serum TNF-α levels and the and the Kyn/Trp value (*r* = 0.67, *p* = 0.000046) in the schizophrenia group.

**Figure 3:**
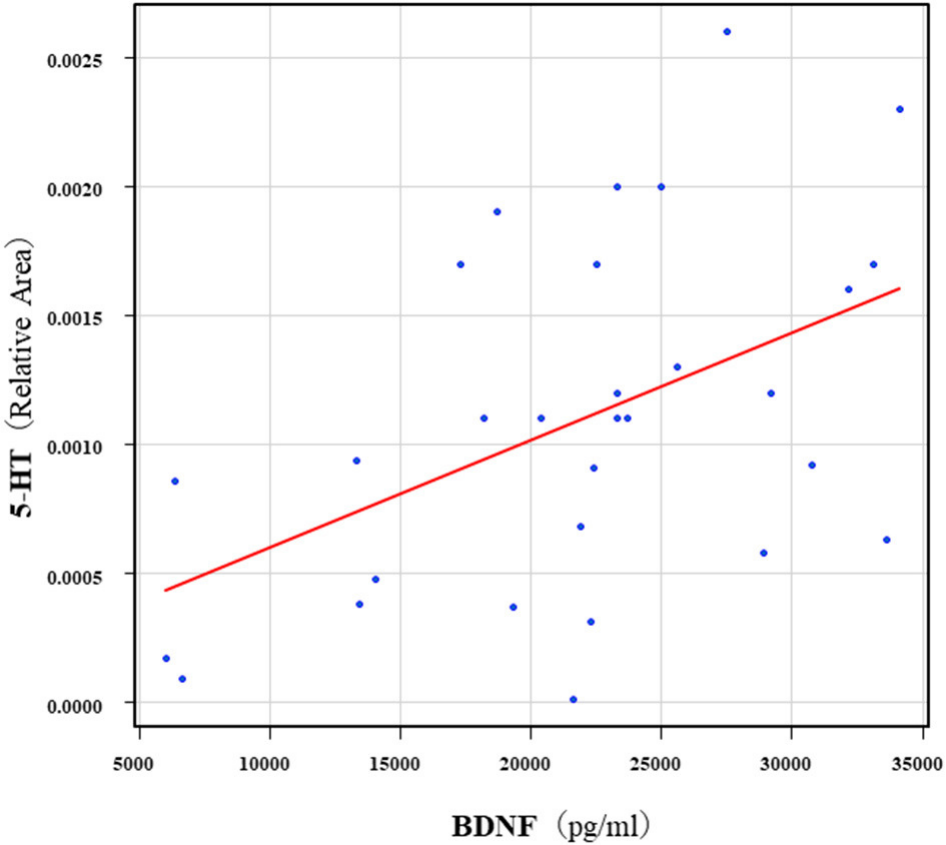
Association between serum 5-HT levels and serum BDNF levels in the schizophrenia group 5. A significant positive correlation was found between the serum BDNF level and the serum 5-HT levels (*r* = 0.49, *p* = 0.0061) in the schizophrenia group. Relative Area: The peak areas of the metabolites were normalized against those of the internal standards, and the resultant relative areas were further normalized by the sample amount explained in the Measurement of metabolites of the Kyn pathway. Thus, serum 5-HT levels demonstrated as the ratio comparing with internal standard.

**Figure 4:**
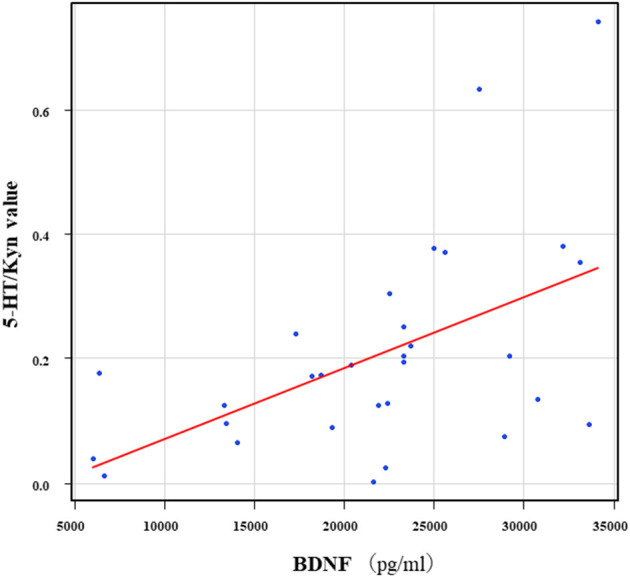
Association between the serum BDNF levels and the 5-HT/Kyn value in the schizophrenia group. A significant positive correlation was found between the serum BDNF levels and the and the 5-HT/Trp value (*r* = 0.55, *p* = 0.0018) in the schizophrenia group.

**Figure 5:**
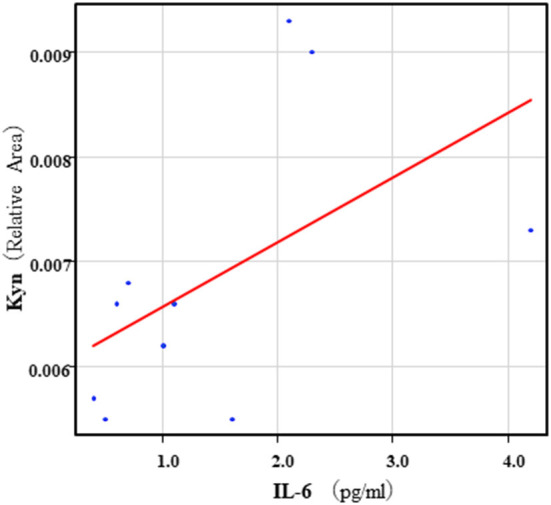
Association between serum IL-6 levels and serum Kyn levels in the healthy control group. A positive correlation was found between the serum IL-6 levels and the serum Kyn levels (*r* = 0.64, *p* = 0.046) in the healthy control group.

**Figure 6:**
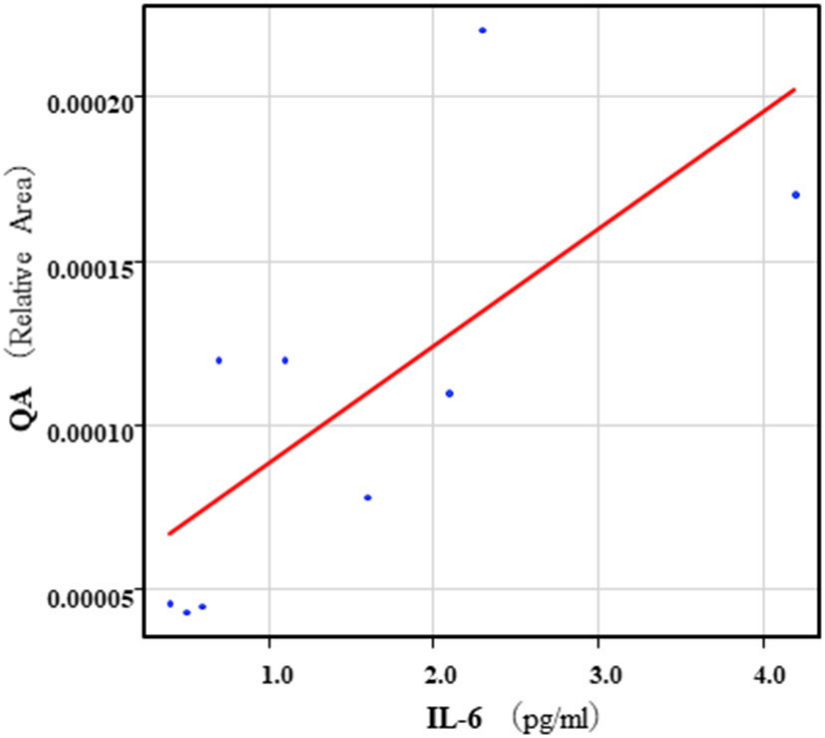
Association between serum IL-6 levels and serum QA levels in the healthy control group. A positive correlation was found between the serum IL-6 levels and the serum QA levels (*r* = 0.78, *p* = 0.010) in the healthy control group. Relative Area: The peak areas of the metabolites were normalized against those of the internal standards, and the resultant relative areas were further normalized by the sample amount explained in the Measurement of metabolites of the Kyn pathway. Thus, serum QA levels demonstrated as the ratio comparing with internal standard.

**Figure 7:**
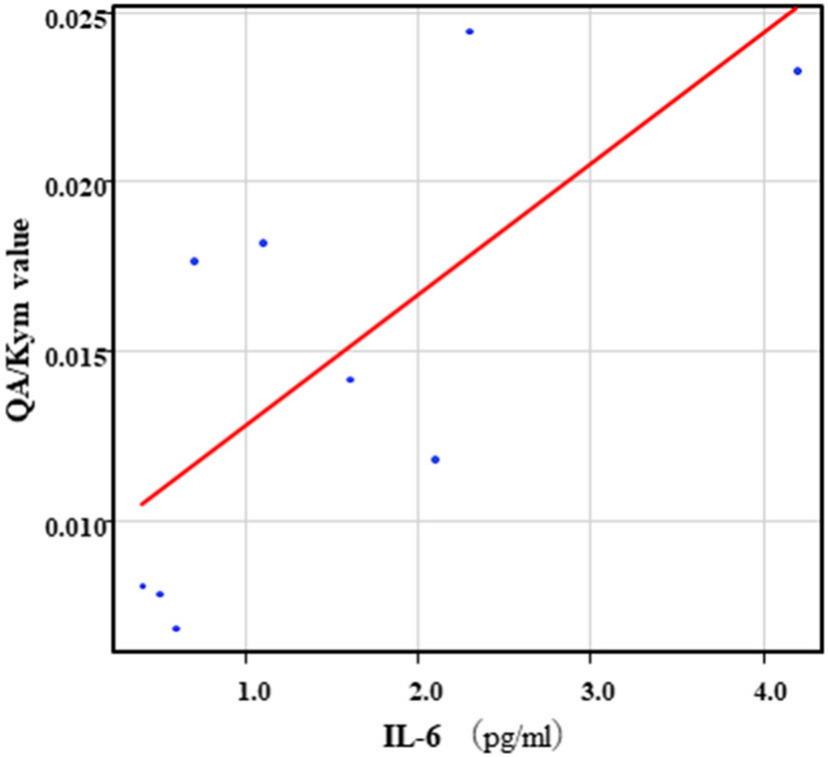
Association between serum IL-6 levels and serum QA/Kyn value in the healthy control group. A positive correlation was found between the serum levels of IL-6 and the serum levels of QA/Kyn value (*r* = 0.77, *p* = 0.021) in the healthy control group.

The correlation table showed the relationships between the Kyn pathway metabolites and the cytokines (TNF-α, IL-6), hsCRP, and BDNF in the schizophrenia group and the healthy control group (Table 4). A significant positive correlation was found between the serum TNF-α levels and the serum Kyn levels (*r* = 0.53, *p* = 0.0026, Figure 1) and the Kyn/Trp value (*r* = 0.67, *p* = 0.000046, Figure 2) in the schizophrenia group. A significant positive correlation was found between the serum level of BDNF and the serum level of 5-HT (*r* = 0.49, *p* = 0.0061, Figure 3) and the 5-HT/Trp value (*r* = 0.55, *p* = 0.0018, Figure 4) in the schizophrenia group (Table 4A). A positive correlation was found between the serum levels of IL-6 and the serum levels of Kyn (*r* = 0.64, *p* = 0.046, Figure 5) and QA (*r* = 0.78, *p* = 0.010, Figure 6) as well as the QA/Kyn value (*r* = 0.77, *p* = 0.021, Figure 7) in the healthy control group (Table 4B).

**Table 4A:** Relationship between metabolites of the Kyn pathway and cytokines, hsCRP, and BDNF in the schizophrenia group.

	**IL-6**	**TNF-α**	**hsCRP**	**BDNF**
Trp	0.028	−0.092	0.33	0.087
	(*p* = 0.89)	(*p* = 0. 63)	(*p* = 0.080)	(*p* = 0.65)
Kyn	0.3	0.53	0.36	−0.21
	(*p* = 0.11)	(***p*** **=** **0.0026**)	(*p* = 0.051)	(*p* = 0.26)
3-HK	0.24	0.29	0.25	−0.21
	(*p* = 0.21)	(*p* = 0.13)	(*p* = 0.18)	(*p* = 0.28)
QA	0.42	0.34	0.27	−0.23
	(*p* = 0.059)	(*p* = 0.12)	(*p* = 0.24)	(*p* = 0.31)
5-HT	−0.3	−0.032	−0.23	0.49
	(*p* = 0.13)	(*p* = 0.87)	(*p* = 0.23)	(***p*** **=** **0.0061**)
Kyn/Trp	0.27	0.67	0.18	−0.26
	(*p* = 0.16)	(***p*** **=** **0.000046**)	(*p* = 0.36)	(*p* = 0.16)
5-HT/Kyn	−0.34	0.1	−0.24	0.55
	(*p* = 0.069)	(*p* = 0.60)	(*p* = 0.22)	(***p*** **=** **0.0018**)
3-HK/Kyn	0.063	0.015	0.1	−0.12
	(*p* = 0.75)	(*p* = 0.93)	(*p* = 0.60)	(*p* = 0.52)
QA/Kyn	0.32	0.25	0.16	0.0085
	(*p* = 0.16)	(*p* = 0.27)	(*p* = 0.50)	(*p* = 0.97)

**Table 4B:** Relationship between metabolites of the Kyn pathway and cytokines, hsCRP, and BDNF in the healthy control group.

	**IL-6**	**TNF-α**	**hsCRP**	**BDNF**
Trp	−0.12	0.25	0.28	0.056
	(*p* = 0.74)	(*p* = 0.49)	(*p* = 0.44)	(*p* = 0.87)
Kyn	0.64	0.61	0.33	−0.25
	(***p*** **=** **0.046**)	(*p* = 0.060)	(*p* = 0.35)	(*p* = 0.49)
3-HK	−0.055	−0.15	0.091	0.29
	(*p* = 0.89)	(*p* = 0.68)	(*p* = 0.81)	(*p* = 0.42)
QA	0.78	0.69	0.58	−0.071
	(***p*** **=** **0.010**)	(*p* = 0.040)	(*p* = 0.10)	(*p* = 0.86)
5-HT	−0.33	0.45	0.46	0.073
	(*p* = 0.35)	(*p* = 0.20)	(*p* = 0.18)	(*p* = 0.84)
Kyn/Trp	0.55	0.46	0.22	−0.18
	(*p* = 0.10)	(*p* = 0.18)	(*p* = 0.53)	(*p* = 0.62)
5-HT/Kyn	−0.35	0.37	0.50	−0.054
	(*p* = 0.33)	(*p* = 0.30)	(*p* = 0.15)	(*p* = 0.88)
3-HK/Kyn	−0.055	−0.15	0.091	0.29
	(*p* = 0.89)	(*p* = 0.68)	(*p* = 0.81)	(*p* = 0.42)
QA/Kyn	0.77	0.63	0.58	−0.075
	(***p*** **=** **0.021**)	(*p* = 0.070)	(*p* = 0.11)	(*p* = 0.84)

*hsCRP, high-sensitivity C-reactive protein; BDNF, brain-derived neurotrophic factor; IL, interleukin; TNF-α, tumor necrosis factor alpha; Trp, tryptophan; Kyn, kynurenine; 3-HK, 3-hydroxykynurenine; QA, quinolinic acid; 5-HT, 5-hydroxytryptamine; KynA, kynurenic acid*.

*Statistically significant differences at P <0.05 is in bold. Positive correlations are shown in red, negative correlations are shown in blue, and the strength of the correlation is expressed in terms of concentration*.

### Relationship Between the PANSS Scores and the Serum Levels of Metabolites of the Kyn Pathway, Cytokines, hsCRP, and BDNF in the Schizophrenia Group

**Figure 8:**
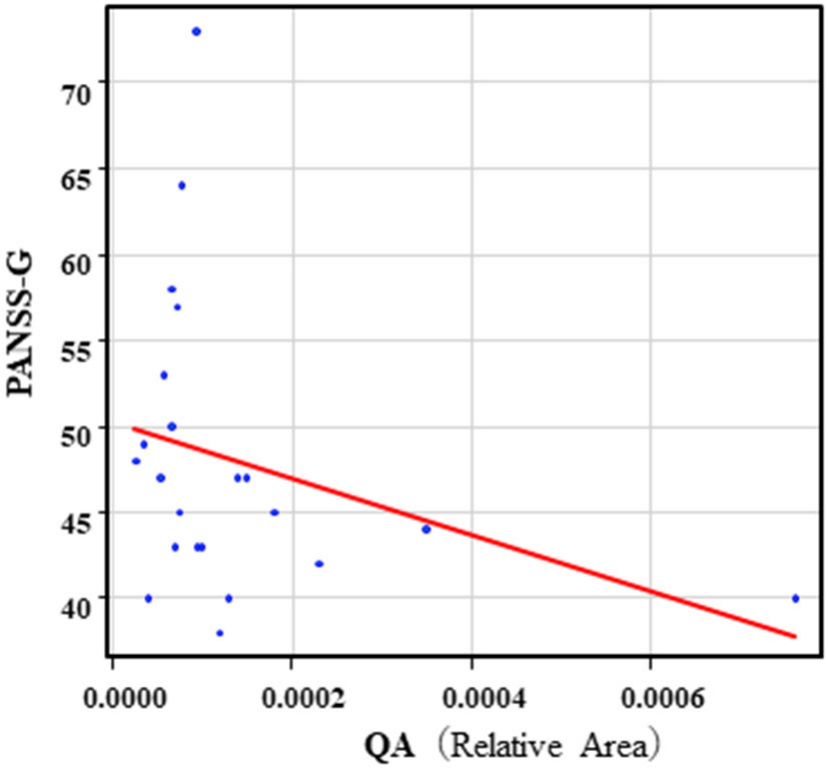
Association between the PANSS-G scores and serum QA levels in the schizophrenia group. A negative correlation was found between the PANSS-G score and serum QA levels (*r* = −0.44, *p* = 0.043) in the schizophrenia group. Relative Area: The peak areas of the metabolites were normalized against those of the internal standards, and the resultant relative areas were further normalized by the sample amount explained in the Measurement of metabolites of the Kyn pathway. Thus, serum QA levels demonstrated as the ratio comparing with internal standard.

**Figure 9:**
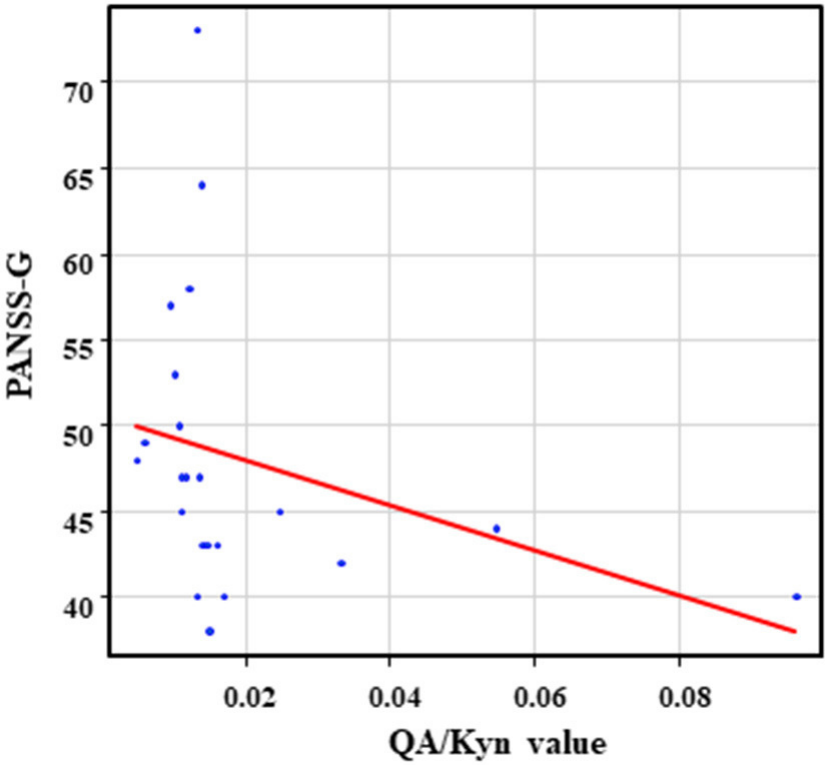
Association between the PANSS-G scores and the QA/Kyn value in the schizophrenia group. A negative correlation was found between the PANSS-G score and the QA/Kyn value (*r* = −0.64, *p* = 0.0014) in the schizophrenia group.

**Figure 10:**
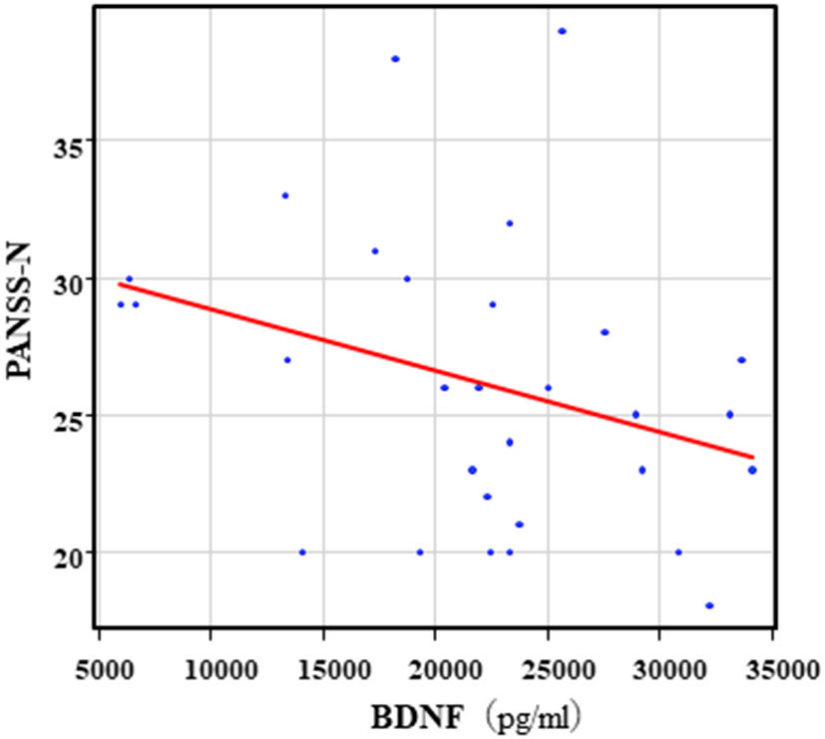
Association between the PANSS-N scores and serum BDNF levels in the schizophrenia group. A negative correlation was found between he PANSS-N score and the serum BDNF levels (*r* = −0.38, *p* = 0.038) in the schizophrenia group.

**Figure 11:**
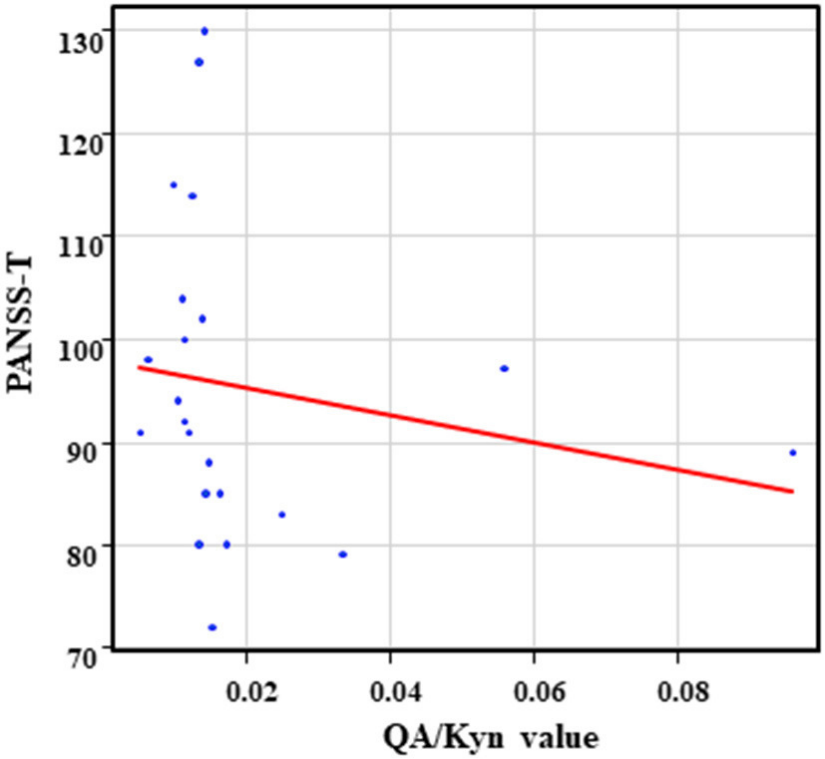
Association between the PANSS-T scores and the QA/Kyn value in the schizophrenia group. A negative correlation was found between the PANSS-T sore and the QA/Kyn value (*r* = −0.50, *p* = 0.017) in the schizophrenia group.

The correlation table showed the relationship between the PANSS scores and the serum levels of metabolites of the Kyn pathway and BDNF (Table 5). A negative correlation was found between the PANSS-G score and the serum QA levels (*r* = −0.44, *p* = 0.043, Figure 8) and the QA/Kyn value (*r* = −0.64, *p* = 0.0014, Figure 9). A negative correlation was found between the serum level of BDNF and the PANSS-N score (*r* = −0.38, *p* = 0.038, Figure 10). A negative correlation was found between the PANSS-T score and the QA/Kyn value (*r* = −0.50, *p* = 0.017, Figure 11).

**TABLE 5:** Relationship between metabolites of the Kyn pathway and positive and negative syndrome scale scores.

	**PANSS-T**	**PANSS-P**	**PANSS-N**	**PANSS-G**
Trp	−0.16	0.0021	0.0019	−0.35
	(*p* = 0.40)	(*p* = 0.99)	(*p* = 0.99)	(*p* = 0.06)
Kyn	−0.17	−0.054	−0.039	−0.28
	(*p* = 0.38)	(*p* = 0.78)	(*p* = 0.84)	(*p* = 0.14)
3-HK	−0.08	0.12	−0.044	−0.13
	(*p* = 0.68)	(*p* = 0.53)	(*p* = 0.82)	(*p* = 0.50)
QA	−0.3	−0.21	−0.15	−0.44
	(*p* = 0.16)	(*p* = 0.35)	(*p* = 0.45)	(***p*** **=** **0.043**)
5-HT	0.19	0.099	0.1	0.18
	(*p* = 0.33)	(*p* = 0.60)	(*p* = 0.58)	(*p* = 0.33)
Kyn/Trp	−0.025	−0.0078	−0.028	−0.0045
	(*p* = 0.89)	(*p* = 0.97)	(*p* = 0.88)	(*p* = 0.98)
5HT/Kyn	0.22	0.13	0.067	0.27
	(*p* = 0.25)	(*p* = 0.51)	(*p* = 0.73)	(*p* = 0.14)
3-HK/Kyn	0.078	0.27	0.014	0.048
	(*p* = 0.68)	(*p* = 0.15)	(*p* = 0.94)	(*p* = 0.80)
QA/Kyn	−0.5	−0.38	−0.24	−0.64
	(***p*** **=** **0.017**)	(*p* = 0.076)	(*p* = 0.28)	(***p*** **=** **0.0014**)
IL-6	−0.33	−0.2	−0.28	−0.29
	(*p* = 0.080)	(*p* = 0.30)	(*p* = 0.14)	(*p* = 0.13)
TNF-α	−0.24	−0.2	−0.13	−0.21
	(*p* = 0.21)	(*p* = 0.30)	(*p* = 0.49)	(*p* = 0.26)
hsCRP	−0.26	−0.28	−0.04	−0.29
	(*p* = 0.18)	(*p* = 0.15)	(*p* = 0.84)	(*p* = 0.13)
BDNF	−0.34	−0.21	−0.38	−0.23
	(*p* = 0.064)	(*p* = 0.26)	(***p*** **=** **0.038**)	(*p* = 0.22)

*PANSS, Positive and Negative Syndrome Scale; PANSS-T, PANSS Total; PANSS-P, PANSS Positive; PANSS-N, PANSS Negative; PANSS-G, PANSS General; Trp, tryptophan; Kyn, kynurenine; 3-HK, 3-hydroxykynurenine; QA, quinolinic acid; 5-HT, 5-hydroxytryptamine; KynA, kynurenic acid; hsCRP, high-sensitivity C-reactive protein; BDNF, brain-derived neurotrophic factor; IL, interleukin; TNF-α, tumor necrosis factor alpha. Statistically significant differences at P <0.05 is in bold. Positive correlations are shown in red, negative correlations are shown in blue, and the strength of the correlation is expressed in terms of concentration*.

## Discussion

Our findings showed a significant positive correlation between the serum levels of TNF-α and the Kyn levels and the Kyn/Trp value in the schizophrenia group, but not in the healthy control group. These results suggest that TNF-α accelerates the formation of Kyn from Trp in patients with schizophrenia. Inflammatory cytokines, especially TNF-α and IL-6, are the primary molecular targets for schizophrenia. Within central nervous system, microglia, the enzyme indoleamine 2,3-dioxygenase plays a role in the metabolism of Trp to Kyn and the subsequent conversion of Kyn to neurotoxic QA. At the same time, cytokine activation shunts metabolic activity from Trp to the Kyn pathway, which further reduces tryptophan hydroxylase driven serotonin synthesis (23). Serum levels of TNF-α in the schizophrenia group were not different from those in the healthy control group in the present study. Thus, it is possible that TNF-α, but not IL-6 more potently activates indoleamine 2,3-dioxygenase and tryptophan 2,3-dioxygenas, two major rate-limiting enzymes of Kyn formation in the schizophrenia group than in the healthy control group. Exposure to chronic mild stress (24), or hepatic encephalopathy (25) has been shown to increase TNF-α and indoleamine 2,3-dioxygenase activity in rats. The serum levels of TNF-α are elevated in schizophrenia (6, 26). Serum levels of IL-6 (26–28), but not the cerebrospinal fluid levels of IL-6 (29), were also elevated in schizophrenia patients. Serum levels of QA were also elevated in psychiatric controls (30). QA, an endogenous metabolite of the Kyn pathway, is toxic and is involved in several neuropsychiatric diseases, including schizophrenia (7, 31). Taken together, these results suggest that IL-6 combined with QA might contribute to neuronal damage in the brain in schizophrenia. A recent systematic review (3) examined the correlation between cytokines and Kyn metabolites, and three studies showed a relationship between the Kyn pathway and elevated IL-6 and TNF-α concentrations. Only one study showed correlations between IL-8 concentrations and the Kyn pathway, and two studies showed correlations of low IL-4 concentrations with the Kyn pathway. Moreover, the authors of the systematic review did not find significant correlations of CRP (*n* = 1 study) and IFN-γ (*n* = 3 studies) with the Kyn pathway in schizophrenia. Meta-analyses of CRP levels in schizophrenia, which included a total of 26 cross-sectional or longitudinal studies evaluating 85,000 participants demonstrated that CRP levels were moderately increased (7). We found a positive correlation between serum levels of hsCRP and serum levels of Kyn in the schizophrenia group. Furthermore, a positive correlation was found between serum levels of BDNF and serum levels of 5-HT in the schizophrenia group, but not in the healthy control group. The 5-HT and BDNF identified in the serum were mainly secreted by platelets (32, 33). The discrepancies between the schizophrenia and healthy control groups might reflect differences in the secretory activity of platelets in both groups. In other words, the interaction between BDNF and 5-HT may be tighter in the schizophrenia group. However, the precise reasons underlying these results remain unknown.

A negative correlation was found between PANSS scores and the serum levels of QA and the QA/Kyn ratio in the schizophrenia group. However, when QA has neurotoxic effects, a positive correlation has been reported be found between PANSS scores and the QA level or QA/Kyn ratio. This discrepancy in the present results was difficult to interpret. QA shows cellular neurotoxicity and has implications in schizophrenia. It has been speculated an imbalance in the production or removal of either of Kyn and QA would be expected to have profound implications for brain function, especially if that imbalance were present chronically (34). Recent study reviewed the roles of imbalances in Kyn metabolism in association with interactions with other neurochemicals as a major contributing pathophysiological mechanism in schizophrenia (35). Actually, QA was correlated with BDNF levels and psychiatric symptoms, suggesting that QA may have an indirect effect on psychiatric symptoms through BDNF but may also have a direct effect simultaneously. This may be biologically plausible because QA is an NMDA receptor agonist that can additionally inhibit the reuptake of glutamate by astrocytes, leading to excitotoxicity (7, 31). We also found a positive correlation between serum QA and serum IL-6 levels in the schizophrenia and healthy control groups. Negative correlations were found between the PANSS-T scores and serum levels of IL-6 and between the PANSS-G scores and serum levels of BDNF in the schizophrenia group. A recent meta-analysis demonstrated that peripheral BDNF levels in serum and plasma were moderately reduced in patients with schizophrenia compared with controls and that this decrease was associated with disease duration. The extent of peripheral BDNF level decrease, however, did not correlate with the severity of positive and negative symptoms (4). Serum levels of IL-6 in chronic schizophrenia patients at admission showed a positive correlation with negative scores, and the serum levels of IL-6 in the patients at discharge were positively correlated with positive, negative, and total scores (36). These findings suggest that the association of serum levels of BDNF and IL-6 with psychometric findings might be complicated in schizophrenia patients. Previous studies have reported that the Kyn pathway is promoted by inflammation, which could be related to the pathological organization of many psychiatric disorders, including schizophrenia. BDNF is a protein produced by nerve cells in the brain and plays an important role in nerve cell activity. Several studies have reported serum Kyn levels in schizophrenia; however, there is no consistent view because of the many conflicting results. Our results showed that there was no significant difference in the plasma levels of Kyn pathway metabolites, cytokines, and BDNF between the schizophrenia and healthy control groups. A partial downregulation of the Kyn pathway is observed in schizophrenia patients, especially during acute symptomatic states and in older age, effects that are independent of each other. In contrast, younger and stable or remitted patients display limited to no Kyn metabolite abnormalities. The current meta-analysis illustrates the dynamic nature of Kyn abnormalities. It should be noted that all included studies investigated peripheral Kyn metabolites, which do not necessarily reflect central Kyn metabolite abnormalities in patients with schizophrenia (37). Finally, the blood levels of metabolites of the Kyn pathway, proinflammatory cytokines, hsCRP, and BDNF in patients with chronic schizophrenia remain controversial. Their interactions may also be complicated. The reasons for this discrepancy in the results remain unknown; however, the diversity of schizophrenia patients enrolled in past and current studies is a plausible explanation.

It has been reported that a positive correlation in Kyn, Trp, or Kyn/Trp ratio between serum and CSF (38), On the other hand, a discrepancy existed in cytokines between serum and CSF (39). Finally, the parallel changes in BDNF levels in plasma and CSF indicate that plasma BDNF levels reflect the brain changes in BDNF levels in schizophrenia (40). The definite discrepancy exists in metabolites of Kyn pathway, cytokines, or BDNF between the peripheral and the brain. Taken together, we could not interpret the results in peripheral as in the brain.

It has been speculated that antipsychotic drugs influence the upregulated Kyn pathway in schizophrenia patients (41). In contrast, treatment of haloperidol and clozapine did not affect the levels of brain Kyn or KynA in mice (9). The precise mechanisms how antipsychotic drugs influence the Kyn pathway and merge their efficacies remain unknown, this must be further elucidated.

This study had several limitations. First, the sample size was small, and all patients with schizophrenia were receiving several antipsychotic drugs, which could have affected the serum Kyn and Kyn metabolite levels. Second, we could not measure KynA in the Kyn pathway, IL-10 and IL-1β. Third, Trp is an essential amino acid that is consumed through diet, and aerobatic exercise but this study did not consider nutritional status, and daily activities of the schizophrenia patients.

In conclusion, TNF-α could influence the Kyn pathway in chronic hospitalized patients with schizophrenia. The relationships of metabolites of the Kyn pathway with the levels of proinflammatory cytokines, hsCRP, and BDNF and psychotic symptoms in chronic schizophrenia are complicated and must be further elucidated.

## Data Availability Statement

The original contributions presented in the study are included in the article/supplementary material, further inquiries can be directed to the corresponding author.

## Ethics Statement

The studies involving human participants were reviewed and approved by Ethics Committee of the University of Occupational and Environmental Health (Approval Number: UOEHCRB19-024). The patients/participants provided their written informed consent to participate in this study.

## Author Contributions

NO and RY: conceptualization. NO: methodology, software, and visualization. NO, YK, AI, and RY: validation and writing—original draft preparation. NO, HT, TN, RI, YK, and AI: data curation. AI, YK, and RY: writing—review, editing, and supervision. RY: funding acquisition. All authors have read and agreed to the published version of the manuscript.

## Conflict of Interest

The authors declare that the research was conducted in the absence of any commercial or financial relationships that could be construed as a potential conflict of interest.

## Publisher's Note

All claims expressed in this article are solely those of the authors and do not necessarily represent those of their affiliated organizations, or those of the publisher, the editors and the reviewers. Any product that may be evaluated in this article, or claim that may be made by its manufacturer, is not guaranteed or endorsed by the publisher.
